# Quantitative analysis of the therapeutic effect of magnolol on MPTP-induced mouse model of Parkinson’s disease using in vivo ^18^F-9-fluoropropyl-(+)-dihydrotetrabenazine PET imaging

**DOI:** 10.1371/journal.pone.0173503

**Published:** 2017-03-03

**Authors:** Chi-Chang Weng, Zi-An Chen, Ko-Ting Chao, Ting-Wei Ee, Kun-Ju Lin, Ming-Huan Chan, Ing-Tsung Hsiao, Tzu-Chen Yen, Mei-Ping Kung, Ching-Han Hsu, Shiaw-Pyng Wey

**Affiliations:** 1 Department of Medical Imaging and Radiological Sciences, Chang Gung University, Taoyuan, Taiwan; 2 Department of Biomedical Engineering and Environmental Sciences, National Tsing Hua University, Hsinchu, Taiwan; 3 Center for Advanced Molecular Imaging and Translation, Department of Nuclear Medicine, Chang Gung Memorial Hospital, Linkou, Taoyuan, Taiwan; 4 Neuroscience Research Center, Chang Gung Memorial Hospital, Linkou, Taoyuan, Taiwan; 5 Institute of Neuroscience, National Chengchi University, Taipei, Taiwan; 6 Department of Radiology, University of Pennsylvania, Philadelphia, Pennsylvania, United States of America; 7 Institute of Radiological Research, Chang Gung University and Chang Gung Memorial Hospital, Taoyuan, Taiwan; University of Chicago, UNITED STATES

## Abstract

^18^F-9-Fluoropropyl-(+)-dihydrotetrabenazine [^18^F-FP-(+)-DTBZ] positron emission tomography (PET) has been shown to detect dopaminergic neuron loss associated with Parkinson’s disease (PD) in human and neurotoxin-induced animal models. A polyphenol compound, magnolol, was recently proposed as having a potentially restorative effect in 1-methyl-4-phenyl-1,2,3,6-tetrahydropyridine (MPTP)- or 6-hydroxydopamine-treated animal models. In this study, ^18^F-FP-(+)-DTBZ PET was used to determine the therapeutic efficacy of magnolol in an MPTP–PD mouse model that was prepared by giving an intraperitoneally (i.p.) daily dose of 25 mg/kg MPTP to male C57BL/6 mice for 5 consecutive days. Twenty-minute static ^18^F-FP-(+)-DTBZ PET scans were performed before MPTP treatment and 5 days after the termination of MPTP treatment to set up the baseline control. Half of the MPTP-treated mice then received a daily dose of magnolol (10 mg/kg dissolved in corn oil, i.p.) for 6 days. ^18^F-FP-(+)-DTBZ PET imaging was performed the day after the final treatment. All ^18^F-FP-(+)-DTBZ PET images were analysed and the specific uptake ratio (SUr) was calculated. Ex vivo autoradiography (ARG) and corresponding immunohistochemistry (IHC) studies were conducted to confirm the distribution of dopaminergic terminals in the striatum. The striatal SUr ratios of ^18^F-FP-(+)-DTBZ PET images for the Sham, the MPTP, and the MPTP + Magnolol-treated groups were 1.25 ± 0.05, 0.75 ± 0.06, and 1.00 ± 0.11, respectively (n = 4 for each group). The ex vivo ^18^F-FP-(+)-DTBZ ARG and IHC results correlated favourably with the PET imaging results. ^18^F-FP-(+)-DTBZ PET imaging suggested that magnolol post-treatment may reverse the neuronal damage in the MPTP-lesioned PD mice. In vivo imaging of the striatal vesicular monoamine transporter type 2 (VMAT2) distribution using ^18^F-FP-(+)-DTBZ animal PET is a useful method to evaluate the efficacy of therapeutic drugs i.e., magnolol, for the management of PD.

## Introduction

Parkinson’s disease (PD) is the second most common neurodegenerative disease worldwide next to Alzheimer’s disease. The typical clinical symptoms of PD are resting tremors, bradykinesia, rigidity, and postural instability. The characteristic pathology of PD is the severe degeneration of dopamine neurons and the depletion of dopamine within the brain [1]. The pathogenesis of PD remains unclear but is likely linked to environmental or genetic factors that contribute to dopaminergic neuron degeneration. Some studies have suggested that oxidative stress, inflammation, mitochondrial dysfunction, and excitotoxicity are related to the pathogenesis of PD [2, 3]. Numerous animal models have been developed to investigate the pathogenesis of PD and to evaluate the efficacy of new treatment modalities against the disease [4–11]. Treating animals with 1-methyl-4-phenyl-1,2,3,6-tetrahydropyridine (MPTP), a neurotoxin that causes selective damage to dopaminergic pathways in a pattern similar to that observed in patients with PD [12], is a commonly used PD model with advantages such as easy manipulation (intraperitoneal injection) and lesion specificity (dopaminergic neuron lesion).

Despite extensive efforts to develop novel treatment against PD [13–17], the therapeutic drugs currently used (e.g., L-3,4-dihydroxyphenylalanine (L-DOPA) or some dopamine agonists) generally only relieve the clinical symptoms of the disease and do not stop the degeneration [18]. Moreover, when L-DOPA is taken over a long period, the dosage required to maintain a constant level of symptomatic relief increases, which may exacerbate the side effects such as fluctuations and dyskinesia [18]. To halt or reverse the degeneration of dopaminergic neurons, new therapeutic drugs are urgently required.

Magnolol, a natural polyphenol extracted from *Magnolia officinalis* or *Magnolia grandiflora*, has been found to suppress the inflammation, oxidative stress, and excitotoxicity of damaged neuron cells [19–23]. In one study, oral magnolol decreased the rate of neuron loss in the hippocampus of senescence-accelerated mice (SAMP1), which was revealed using Bodian’s staining of brain sections [19]. Elsewhere, activity assays on mitochondria and lactate dehydrogenase revealed the antioxidizing effect of Magnolol in rat granule cells [20]. Magnolol treatment also prevented SAMP8 mice from experiencing memory loss and prevented a reduction in their cerebral cholinergic neurons, as demonstrated by behaviour tests, subsequent immunohistochemistry (IHC) staining of choline acetyltransferase-positive cells, immunoblots of Akt phosphorylation, and thiobarbituric acid reactive substance assay [21]. Despite these promising studies, no in vivo imaging study investigating the protective effect of magnolol on neurodegenerative animal models has yet been conducted. An apomorphine-induced rotation test and tyrosine hydroxylase (TH) assay (immunoblot and IHC) previously revealed the protective effect of magnolol on dopaminergic neurons in 6-hydroxydopamine- (6-OHDA-) lesioned mice [22]. Immunoblots of the dopamine transporter (DAT), TH and glial fibrillary acidic proteins demonstrated that magnolol prevented striatal lipid peroxidation in MPTP-treated mice, and decreased 1-methyl-4-phenylpyridinium- (MPP^+^-) induced cytotoxicity and the formation of reactive oxygen species in human neuroblastoma cells [23].

In the last decade, numerous radiotracers for imaging with positron emission tomography (PET) have been developed that target different binding sites within presynaptic dopamine neurons. For imaging vesicular monoamine transporter type 2 (VMAT2), a protein responsible for packing monoamine neurotransmitters from the cytoplasm into the vesicles for storage and subsequent synaptic release, ^11^C-(+)-dihydrotetrabenazine [^11^C-(+)-DTBZ] has been used as a PET tracer with suitable binding properties and specificity [24–27]. Because more than 90% of VMAT2s are located in the dopamine nerve terminals [28], in vivo estimation of VMAT2 density using ^11^C-(+)-DTBZ PET has attracted considerable interest for the diagnosis and management of PD and associated nigrostriatal degeneration disorders. However, the short physical half-life of ^11^C (20 min) limits the use of ^11^C-(+)-DTBZ to PET centers equipped with a cyclotron/radiochemistry facility and restricts further clinical applications of the tracer. This problem was overcome through the development of ^18^F-9-fluoropropyl-(+)-dihydrotetrabenazine [^18^F-FP-(+)-DTBZ], a ^18^F-labelled derivative of ^11^C-(+)-DTBZ with a longer physical half-life (109 min) [29]. The performance of ^18^F-FP-(+)-DTBZ as a superior PET tracer for VMAT2 imaging has been verified in animal models [30, 31] as well as in clinical studies with PD patients [32–36].

The purpose of this study was to monitor the therapeutic response of magnolol treatment on MPTP-intoxicated animal models of PD using in vivo ^18^F-FP-(+)-DTBZ PET imaging. This combined method advances the possibility of lower costs for new drug screening for PD treatment and an expedited development of appropriate medications.

## Materials and methods

### Animals

Male C57BL/6 mice (5–8 weeks old, 25–35 g) were housed in clear cages in a controlled environment (temperature 22–25°C) and allowed to acclimate for at least one week with food and water available ad libitum. A 12-h light–dark cycle was maintained. The procedures for the care of the animals and the following experiments were approved by the Institutional Animal Care and Use Committee at Chang Gung University and Chang Gung Memorial Hospital (Taoyuan, Taiwan), respectively.

### Experimental design

**Fig 1:**
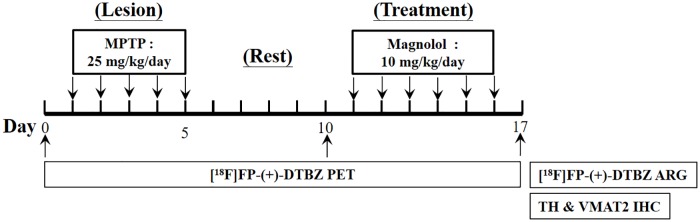
Experimental paradigm of therapeutic effect of magnolol on MPTP-lesioned animals.

Details of the experimental design and information regarding the animal groups in this study are presented in Fig 1. Twelve mice were first randomly assigned for MPTP treatment and the Sham control (n = 8 and 4, respectively). The mice for MPTP treatment were intraperitoneally (i.p.) injected with a single daily dose of 25 mg/kg MPTP hydrochloride (Sigma–Aldrich, St. Louis, MO, USA) solution for 5 consecutive days (days 1–5) [37]. By contrast, only saline (the vehicle of MPTP) was administered i.p. to the mice of the Sham group, following the same protocol as for the MPTP group.

Magnolol (Sigma–Aldrich, St. Louis, MO, USA) dissolved in corn oil (10 mL/kg) was administerted i.p. with a single daily dose of 10 mg/kg to four MPTP-lesioned mice (further assigned as the MPTP + Magnolol group), 6 days after the final MPTP treatment for 6 consecutive days (days 11–16). Another four MPTP-treated mice served as the lesion control (designated as the MPTP group), and together with the Sham group were injected i.p. with corn oil only, following the same protocol as for the MPTP + Magnolol group.

Each animal received three ^18^F-FP-(+)-DTBZ PET scans. A baseline PET scan was conducted one day before MPTP treatment (day 0), a second PET scan was performed one day before magnolol treatment (day 10), and a third PET scan was conducted on the day following the final magnolol treatment (day 17). Immediately after the third PET scan, the animal brains were dissected for further autoradiographical and immunohistochemical studies.

### Radiochemistry

Optically pure ^18^F-FP-(+)-DTBZ was prepared at the cyclotron facility of Chang Gung Memorial Hospital (Taoyuan, Taiwan) using the precursor AV-244 obtained from Avid Radiopharmaceuticals (Philadelphia, PA, USA). The radiochemical purity of the ^18^F-FP-(+)-DTBZ was greater than 98%, and the specific activity was 60–200 TBq/mmol at the end of synthesis [33].

### PET scan and image analysis

The small animal ^18^F-FP-(+)-DTBZ PET imaging followed a previously published protocol [38], and all PET images were acquired on a preclinical Inveon PET system (Siemens Medical Solutions, Knoxville, TN, USA). Each mouse was subjected to a 20-min static scan under isoflurane anesthesia (1.5% in oxygen gas) 30 min after receiving a single bolus injection of ^18^F-FP-(+)-DTBZ (22.76 ± 0.45 MBq in 0.1 mL saline) through a tail vein.

All image data were processed and analysed using PMOD image analysis software (v. 3.2; PMOD Technologies, Zurich, Switzerland). Each image was normalised according to the corresponding ^18^F-FP-(+)-DTBZ PET mouse template [39] and coregistered to the PMOD built-in T2-weighted magnetic resonance imaging (MRI) template. The built-in volume of interests (VOIs) of the striatum and the cerebellum from the MRI template were applied to normalised PET images for quantification. The striatal specific uptake ratio (SUr) was calculated as [(uptake in striatum − uptake in cerebellum)/ (uptake in cerebellum)], where the cerebellum was used as the reference region [38].

### Ex vivo autoradiography studies

Quantitative ^18^F-FP-(+)-DTBZ autoradiography (ARG) analysis was performed for 9 animals (3 for each group) immediately after completion of the third PET scan (day 17) using a protocol described previously [40]. The mice were euthanised through cervical dislocation, and the brains were immediately removed and frozen on dry ice. Coronal sections of 20-μm thickness were cut on a cryostat microtome (CM3050S; Leica, Bensheim, Germany). Subsequently, the dried sections were exposed overnight to an imaging plate (BAS SR2040, 20 × 40 cm^2^; Fujifilm, Tokyo, Japan). The imaging plate was then scanned with a phosphor image reader (FLA-5100; Fujifilm, Tokyo, Japan), and the obtained images were analysed using Multi Gauge 3.0 software (Fujifilm, Tokyo, Japan) to calculate the striatal specific uptake ratio (SUr), which is defined as (PSL_striatum_ − PSL_cerebellum_)/PSL_cerebellum_ [41], where PSL is the photostimulated-luminescence intensity [42, 43]. Five consecutive brain sections from each animal were used to determine the target PSL.

### Immunohistochemical studies

To confirm the selective dopaminergic neuron damage among the three animal groups, striatal sections (20-μm thickness) adjacent to the ARG sections were removed and stained with rabbit anti-TH and rabbit anti-VMAT2 antibodies. The DAB (3, 3’-diaminobenzidine) staining was performed using an UltraVision Quanto Detection System HRP DAB kit (Thermo Scientific, San Jose, CA, USA). The concentrations of the primary antibodies of TH (AB152; Millipore, Temecula, CA, USA) and VMAT2 (ab81855; Abcam, Cambridge, UK) were 1:200 and 1:2000, respectively, and the antibodies were incubated for 30 min. Upon completion of the staining, photographs were taken using an optical microscope (Eclipse E600; Nikon, Tokyo, Japan) equipped with a digital camera. (DS-Fi1; Nikon, Tokyo, Japan).

### Statistical analysis

All data are expressed as mean ± standard deviation. The differences between groups were determined using one-way analysis of variance followed by the Student–Newman–Keuls post-hoc test. A p value of 0.05 was defined as the threshold of statistical significance in all tests.

## Results

### ^18^F-FP-(+)-DTBZ PET imaging

Representative static PET images (30–50 min after injection, acquired on day 17) of ^18^F-FP-(+)-DTBZ in the brains of the Sham, the MPTP, and the MPTP + Magnolol mice are presented in Fig 2. An intensive and symmetric normal distribution of ^18^F-FP-(+)-DTBZ was clearly evident in the striatum of the Sham mice (Fig 2A), whereas the striatal uptake of ^18^F-FP-(+)-DTBZ detected in the MPTP mice was substantially less obvious (Fig 2B). After six consecutive daily magnolol treatment, the striatal uptake of ^18^F-FP-(+)-DTBZ was substantially higher in the MPTP + Magnolol mice than that in the MPTP-lesioned mice (Fig 2C). In all of the animals, a small and insignificant uptake of ^18^F-FP-(+)-DTBZ was detected in the cerebellum region, which is almost devoid of VMAT2 (Fig 2A, 2B and 2C).

**Fig 2:**
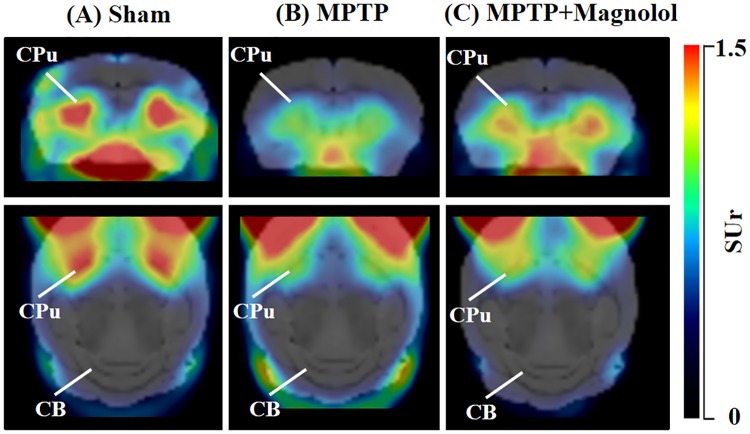
Coregistered ^18^F-FP-(+)-DTBZ PET and T2-weighted MRI representative images in coronal and axial views of C57BL/6 mouse brain. (A) The Sham, (B) the MPTP, and (C) the MPTP + Magnolol groups. Images were acquired one day after the final magnolol treatment (day 17). CPu: caudate putamen; CB: cerebellum.

An analysis of the ^18^F-FP-(+)-DTBZ PET imaging data for the animals in the Sham, the MPTP, and the MPTP + Magnolol (n = 4) groups is presented in Table 1. Notably, the baseline PET images of all 12 mice on day 0 showed a normal striatal SUr of 1.31 ± 0.03.

**Table 1:** Striatal SUr of ^18^F-FP-(+)-DTBZ found by PET studies of the brains of the Sham, the MPTP, and the MPTP + Magnolol group mice (n = 4 in each group).

	Sham	MPTP	MPTP + Magnolol
Day 0 (baseline)	1.31 ± 0.03 (n = 12)		
Day 10	1.27 ± 0.05	0.71 ± 0.03* (n = 8)	
Reduction		−45.80%	
Day 17	1.25 ± 0.06	0.75 ± 0.06*	1.00 ± 0.11*^†^
Reduction		−42.75%	−23.66%

% Reduction was relative to the baseline value.

*p < 0.05 vs. the Sham group;

^†^p < 0.05 vs. the MPTP group.

Five days after MPTP treatment (day 10), the significantly lower striatal SUr was discovered in all MPTP-lesioned mice (0.71 ± 0.03; n = 8; including the MPTP and the MPTP + Magnolol groups before magnolol treatment), compared with the Sham mice (1.27 ± 0.05; n = 4) (p < 0.05). On day 10, there was no significant difference in striatal SUr between the Sham mice and the baseline (1.27 ± 0.05 vs. 1.31 ± 0.03; p > 0.05), thus it excluded the possibility that i.p. injection of saline (the vehicle of MPTP) damaged the dopaminergic neurons.

PET images on day 17, one day after the final magnolol treatment, revealed a significantly higher striatal SUr in the MPTP + Magnolol mice (1.00 ± 0.11; n = 4) than that in the MPTP control group (0.75 ± 0.06) (p < 0.05). Moreover, there was no significant difference between the striatal SUr of the Sham group on days 17 and 10 (1.25 ± 0.06 vs. 1.27 ± 0.05; p > 0.05), which excludes the involvement of the i.p. injection of corn oil (the vehicle of magnolol) in these striatal VMAT2 level changes.

### Ex vivo autoradiography studies

**Fig 3:**
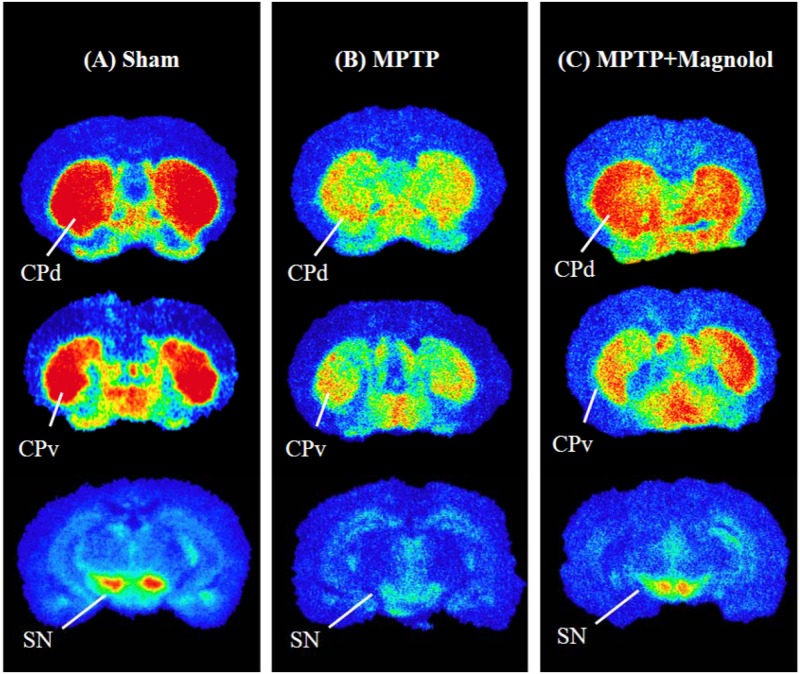
Ex vivo ARG of ^18^F-FP-(+)-DTBZ in C57BL/6 mouse brain. ARG performed immediately after the third PET scan on day 17. (A) The Sham, (B) the MPTP, and (C) the MPTP + Magnolol mice. CPd: dorsal caudate putamen; CPv: ventral caudate putamen; SN: substantial nigra.

To further confirm the PET imaging results and provide anatomic details, ex vivo ARG (Fig 3) was performed immediately after completing the third PET scan. The radioactivity was concentrated in the dopamine-enriched caudate putamen and substantial nigra in the Sham mice (Fig 3A), whereas the significantly reduced labelling of ^18^F-FP-(+)-DTBZ in MPTP-lesioned animals was detected in those regions (Fig 3B). After magnolol treatment, partial recovery of the ^18^F-FP-(+)-DTBZ labelling (Fig 3C) was observed, indicating that magnolol treatment improved the neuronal damage in MPTP-lesioned animal brains.

**Fig 4:**
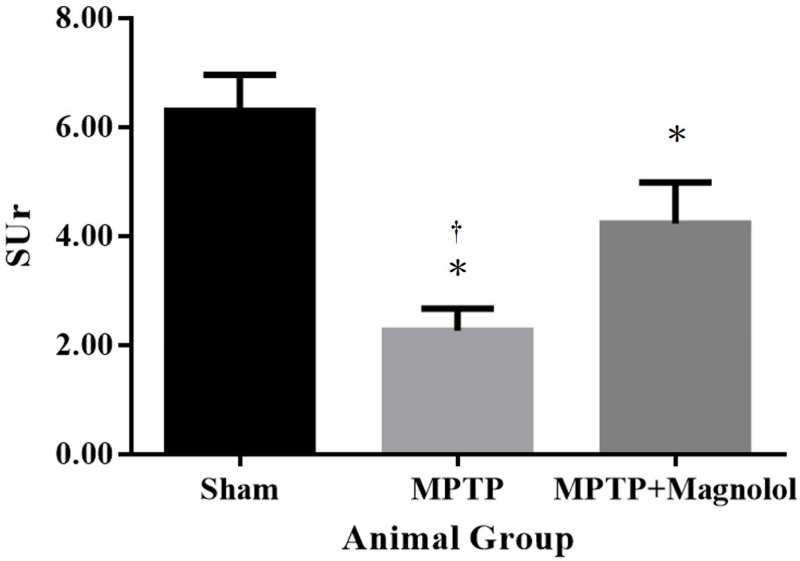
Quantitative analysis of the ex vivo ^18^F-FP-(+)-DTBZ ARG in the Sham, the MPTP, and the MPTP + Magnolol mice (n = 4 in each group). *p < 0.05 vs. the Sham group; †p < 0.05 vs. the MPTP group.

**Fig 5:**
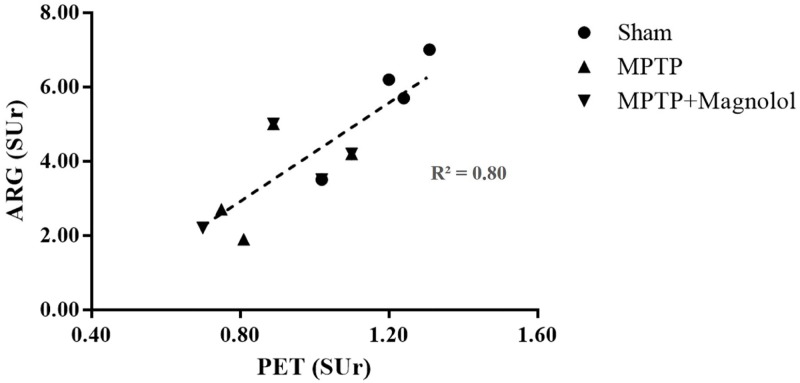
Scatter plot and linear regression analysis between ex vivo ARG data and quantitative striatal ^18^F-FP-(+)-DTBZ SUr data obtained using PET imaging.

Quantitative analysis of the ARG (Fig 4) revealed that the striatal SUr was 6.30 ± 0.66 in the Sham mice. The MPTP-induced lesions significantly reduced the striatal SUr to 2.27 ± 0.40 (p < 0.05 vs. the Sham group). In addition, the striatal SUr in the MPTP + Magnolol mice (4.23 ± 0.75) was significantly higher than that of the MPTP group (p < 0.05). The in vivo VMAT2 estimation was confirmed by ex vivo ARG. Magnolol treatment recovered the decreases in striatal SUr triggered by MPTP intoxication from −64% to −32% relative to the Sham control. The results of the ^18^F-FP-(+)-DTBZ PET imaging correlated favourably with the ex vivo ARG data with a Pearson correlation coefficient of 0.82 (Fig 5).

### Immunohistochemical studies

**Fig 6:**
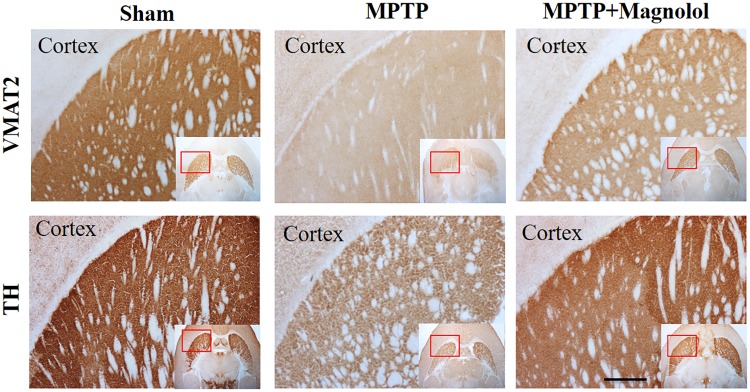
Immunohistochemical staining of anti-VMAT2 and anti-TH antibodies in striatal sections of the Sham, the MPTP, and the MPTP + Magnolol mice. Bar = 400 μm.

Immunohistochemical staining against VMAT2 and tyrosine hydroxylase (TH), two selective presynaptic dopaminergic neuron markers, was conducted to detect the specificity of ^18^F-FP-(+)-DTBZ uptake in the striatum. Magnolol treatment remarkably reversed the reduced expression of striatal VMAT2 and TH in the MPTP-lesioned mice. The data showed that the density of dopaminergic neuron determined by immunohistochemical staining was related to that observed in ^18^F-FP-(+)-DTBZ uptake between study groups (Fig 6).

## Discussion

PET imaging has long been used in the evaluation of PD, primarily as a means to assess presynaptic dopaminergic integrity by targeting DOPA decarboxylase, DAT, and VMAT2. These markers of presynaptic dopaminergic integrity decline following an exponential function that is estimated to deviate from normal age-related changes several years before the onset of clinical PD symptoms. VMAT2 declines before the others [28], and it is less susceptible than DAT or DOPA decarboxylase to compensatory changes during the course of the disease [44–47]. Using recent advances in PET technology that facilitate the imaging of small animals, the use of the VMAT2 ligand ^18^F-FP-(+)-DTBZ to visualise the decline of striatal tracer uptake in MPTP-induced PD mouse models has recently been reported [38,48]. In vivo PET imaging provides the opportunity to explore longitudinal nigrostriatal dopaminergic neuron changes in a particular animal, which could minimise subjective deviations between diverse animal groups.

In the present study, the combination of ^18^F-FP-(+)-DTBZ PET imaging and the MPTP-induced mouse model of PD was used to determine the neuroprotective effect of magnolol on dopaminergic neurons. From the PET imaging data after quantification and calculation, we discovered a substantial reduction of ^18^F-FP-(+)-DTBZ SUr approximately 46% on day 10 in all MPTP-treated animals (n = 8, including the MPTP and the MPTP + Magnolol groups before magnolol treatment). In the MPTP and the MPTP + Magnolol animals (n = 4 for each group), the decreases in striatal ^18^F-FP-(+)-DTBZ SUr on day 17 were approximately 43% and 23%, respectively, compared to the baseline on day 0. This indicated an approximately 22% recovery of striatal ^18^F-FP-(+)-DTBZ SUr in the MPTP + Magnolol mice compared with the MPTP mice. This therapeutic effect of magnolol is consistent with a previous report by Muroyama et al. [23], in which magnolol treatment significantly mitigated MPTP-induced decreases in DAT and TH protein levels (according to the quantification of related Western blotting data).

When using the MPTP-treated mice as a PD animal model, the issue of reproducible neuroplasticity should be noted [49–51]. The time-dependent reproducible return of striatal dopamine is found in MPTP-lesioned mice weeks or months after injury. This surviving nigrostriatal dopaminergic neurons have robust and reproducible neuroplasticity that was previously related to the animals’ TH protein levels [51]. In the present study, the tracer uptake of the MPTP group did increase on day 17 compared with that group’s data from day 10. However, the self-recovery was not statistically significant.

Because of the small sizes of mouse brains and the limited PET image resolution, detailed observation of the subregional contour of the images was difficult. For the PET imaging analysis in the present study, we applied spatial normalisation with a ^18^F-FP-(+)-DTBZ PET template (which had been validated previously [39]) prior to the image quantification analysis. Furthermore, to prevent bias from the selection of manual VOIs, the template’s built-in VOIs were used herein. Using the imaging analysis we discovered that the quantification values of SUr in the present study were smaller than in the literature [38], which may be attributable to the differences in VOI drawing methods.

Ex vivo ARG was performed for all the imaged animal groups to investigate the full tracer distribution and verify the in vivo PET imaging data. Contours were clearly delineated in the ARG images compared with the in vivo PET images. Additionally, the tracer uptake was significantly lower in the dopaminergic neuron-rich areas of the MPTP group compared with the Sham group. After magnolol treatment, the tracer uptake was approximately 22% recovered, which was consistent with the findings of in vivo PET imaging. Both imaging quantification results were favourably correlated; however, we also discovered that the ARG results were more sensitive than the PET results, which is similar to data published elsewhere [38]. This may have been a result of the limited resolution and partial volume effect of the current PET technique, as well as the difference in sample size (i.e., 20 μm for ARG; 0.8 mm for PET) [52].

To further evaluate the therapeutic effects of magnolol on the lesioned dopaminergic neurons induced by MPTP, we performed VMAT2- and TH-IHC on a section adjacent to that used in the ARG. The images obtained resembled those that would be produced through in vivo imaging or ex vivo ARG.

## Conclusions

^18^F-FP-(+)-DTBZ PET imaging can visualise the therapeutic effect of magnolol on the dopaminergic neurons of MPTP-lesioned PD mouse models, while in vivo imaging of striatal VMAT2 distribution using ^18^F-FP-(+)-DTBZ PET can be a valuable method for monitoring and quantifying the treatment efficacy of neuroprotective agents on PD.
